# Putting People in Boxes: Iranian Exiles and Queer Identities

**DOI:** 10.1007/s12119-025-10395-4

**Published:** 2025-06-28

**Authors:** Moira Dustin

**Affiliations:** School of Law, Politics and Sociology, University of Sussex, Brighton and Hove, UK

**Keywords:** Iran, Queer, Exile, Asylum, Identity, SOGIESC

## Abstract

Sexual orientation, gender identity, gender expression and sex characteristics (SOGIESC) are no longer uniformly viewed as stable and permanent individual attributes in academic and public discourse. Iran, and Iranian scholarship, has been an important site for this destabilisation. Postcolonial, feminist and queer theorists have mapped changes since the nineteenth century in how Iranian individuals and society perceive sexual and gender identities and behaviours, often attributing shifts in discourse to Iran’s engagement with Europe. Without overlooking the marginalisation and abuse of queer Iranians, this scholarship undermines simplistic Western narratives to create a more nuanced analysis of their experiences and related discourses. Meanwhile, and in contrast, in the broader field of refugee law and scholarship, homophobia and transphobia are now recognised as grounds for claiming asylum in Western refugee-receiving states, however the criteria for recognition assumes the stable conceptualisations of sexuality and gender identity that have been disrupted in other fields. This article juxtaposes these two bodies of work. Using data from interviews with queer Iranians in exile, I ask whether the contradiction between law’s reliance on categories and the mutability of sexuality and gender as lived is detrimental for queer Iranians seeking international protection, and for asylum claimants in general.

## Introduction

Gender and queer scholarship have effectively undermined binary and static conceptions of sexuality and gender (Butler, 2006; Kosofsky Sedgwick, 2008; Seidman, 1996). In overlapping literature, postcolonial and historical writers on Iran have shown how constructs of sexual and gender identity have developed over time in ways that are specific to Iranian history and its engagement with the outside world (Afary, 2009; Korycki & Nasirzadeh, 2016; Mahdavi, 2012; Najmabadi, 2005; Shakhsari, 2012, 2014). Elsewhere, in the field of refugee law, policy and discourse, there is now recognition that homophobia and transphobia are forms of persecution and therefore the basis for claiming international protection (UNHCR, 2012). However, the extension of protection to queer individuals typically relies on fixed and static identity-based conceptualisations of non-conforming sexual orientation, gender identity, gender expression and sex characteristics (SOGIESC) personhood. Individuals claiming asylum need to demonstrate that they genuinely are gay, lesbian or transgender to establish their credibility, with the assumption that this is a legitimate endeavour because sexuality and gender identity are truths that may be proven in a legal process.

In this article, I consider prevalent approaches to SOGIESC asylum alongside scholarship about SOGIESC in Iran. Using interviews with exiled queer Iranians, I argue that how queer Iranians understand and articulate their sexual and gender identities is at odds with asylum decisionmakers’ expectation that claimants describe a linear psychological journey from ignorance to awareness of queer identity. While there is now some recognition that sexual and gender identities are not discrete and static, the research on which this article is based suggests that there is still a long way to go before refugee law and practice resonates with the narratives of many queer exiled Iranians.

I start with a discussion of the article’s underpinning methodology, including considerations of terminology (Methodology and Terminology). In the section SOGIESC in Iran, I provide an overview of the rich literature exploring and complicating Iranian sexuality and gender and mapping developments over time and in relation to national and international events. In the following section, SOGIESC Refugee Protection, I contrast this complexity with the categorical and category-based nature of refugee law, summarising the development of refugee protection for cases based on sexuality, gender and gender identity-based persecution, including the application of the Particular Social Group Refugee Convention ground. The section after, Voices of queer Iranians in exile, draws on the voices of queer exiled Iranians in Turkey, the UK, and Canada, to illustrate the dynamic nature of identity as experienced by these individuals, indicating the misalignment between queer identities as lived and the imperatives of refugee law. The subsequent part, Asylum Determination and Lived Experience, develops this incongruity further, discussing the contrast between the queer subjects that refugee law recognises and protects, and the queer subjectivities articulated by our project’s Iranian research participants. In the Conclusion, I consider the implications for fair and responsive refugee status determination as the basis for possible further research on improvements to the interpretation of the Refugee Convention.

## Methodology and Terminology

In this article, I draw on fieldwork from the project ‘Negotiating Queer Identities Following Forced Migration: A Comparative Study of Iranian Queer Refugees Living in Turkey, the UK and Canada’ (2022–2024). This mixed-method and interdisciplinary project, funded by the Economic and Social Research Council and based at the University of Sussex, aimed to develop new critical understanding of the processes of identity formation and transformation for queer Iranians in exile in Turkey, the UK and Canada. The project included semi-structured qualitative interviews with 57 individuals based in Turkey, the UK and Canada as well as with a small number of participants interviewed (remotely) in Iran. Participants’ SOGIESC, religious beliefs or non-beliefs, socio-economic and educational backgrounds, and ages were diverse. Some, but not all, were or had been asylum claimants and refugees. This meant their articulations of identity were not only a response to the requirement to mould their identities for asylum purposes but part of a broader discussion about their changing lives and self-perception. The majority of the interviews were carried out in Farsi and transcribed and then translated into English before coding using the NVivo software. This is worth mentioning as, although the project team combined Farsi and English speakers, language and cultural differences emerged as a complicating factor.

The project team received ethical approval from the University of Sussex.^1^ Recognising that many of those interviewed had experienced trauma, including sexual and gender-based violence (SGBV), we took measures to ensure that their participation in the project was a rewarding experience based on informed consent. We invited participants to use a pseudonym of their choice, if they wished, and explained that they were free to withdraw consent at any time prior to publication of outputs. Alongside the interviews and theoretical analysis, the project used creative methods—poetry workshops, films and podcasts—to enhance our participants’ voices and reflect the significance of art and poetry in Iran.^2^

The interdisciplinary nature of the project helps to highlight the contradictions this article explores. A postcolonial lens often complicates what seems a straightforward scenario, and this is certainly the case in relation to Iranian sexuality and gender, as the next section shows. In contrast, law and legal studies typically privilege positivist thinking, and have as their premise an (allegedly) objective and neutral starting point, as identified and problematised by feminist theorists (Brayson, 2019; Lacey, 1995). Similarly, refugee law depends on verifiable truths and clear distinctions between well-founded fear and unfounded fear, and between persons deserving and persons not deserving of protection (Foster & Hathaway, 2014). The subtlety and ambivalence of analyses from postcolonial and feminist literature is often in tension with the requirement for precision and clarity in law and legal studies. The argument here is that this tension is problematic when the complexity of actual experiences fails to correlate with the simplistic narratives needed to secure refugee protection, as I go on to demonstrate.

In interrogating the tension between legal and postcolonial perspectives, an immediate question of terminology arises: how to describe the non-hetero- and non-cis-normative Iranian individuals that are the focus of the article when descriptions are part of the very problem I address. All participants were asked how they identified or wished to identify themselves as part of the interview process, and the responses were diverse, from the more common categories (gay, trans woman), to the less common (pan sexual, homoflexible), to the rejection of any category—a number of participants questioned the need to use labels of any kind. As Jebo said, ‘[t]his categorizing makes me uncomfortable’, a concern that is explored further in this article.

Terminology may also vary depending on whether identity or behaviour is emphasised, with cultural implications. Identity-based terms have historically been more common in Western and European discourse, including asylum and human rights discourse, where the most common descriptors have historically been variations on ‘LGBTQI+’^3^ and more recently, people with ‘diverse SOGIESC’.^4^ Alternatively, and as explored in the next section, SOGIESC identities have long been portrayed as more fluid in Middle-Eastern and Asian countries, including Iran. The project on which this article is based uses the adjective ‘queer’ as an inclusive umbrella term for people who are unable or unwilling to conform to sexual and gender social norms. Yet even this most porous of categories is a category and serves to classify people for a particular purpose. It may be seen as too all-encompassing by some, exclusive of trans identities by others, or rejected for entirely different reasons (Jagose, 1996; Penney, 2014; Stryker, 2004). Hence my recognition that language has significant limitations, and particularly so in a cross-cultural context.

## SOGIESC in Iran

The evolution of sexuality, gender and gender identity in Iran has been extensively explored. Postcolonial and historical scholars have mapped changes since the nineteenth century in how Iranian sexual and gender identities and behaviours are discursively framed, often attributing shifts in discourse to engagement with Europe or ‘Westoxication’ (Hanson’s term), and illustrating the complexity of constructs of sex and gender as experienced by the individual and in relation to changing social mores and law (Afary, 2009; Hanson, 1983; Korycki & Nasirzadeh, 2016; Mahdavi, 2012; Najmabadi, 2005; Shakhsari, 2012, 2014).

Much of this literature describes Iranian sexual and gender identities in ways that do not align with Western or European articulations of LGBTQI+ identities as permanently defined and definable. This includes work that identifies the prevalence and acceptance of same-sex relationships and encounters in Iran dating back centuries (Afary, 2009; Babayan & Najmabadi, 2008; El-Rouayheb, 2005; Floor, 2008; Habib, 2007). Ambiguity about the subject’s gender in classical love poetry is enhanced by the lack of gender in Persian grammar, and this ambiguity is more recently evident in the abundance of Iranian films exploring gender and sexuality in modern Iranian cinema (Vanzan, 2014). At the same time, the internet has become a critical toolbox for Iranians at home and the Iranian diaspora to build online spaces to play with sexuality and gender roles and identities (Rahbari, 2022; Yaghoobi, 2021).

Overlapping with the acceptance of same-sex relations is the understanding of sexuality in terms of behaviour rather than identity, and thus dependent on location and stage of life. Prominent here is the work of Najmabadi. In ‘Professing Selves’, she argues that the sex-gender distinction taken for granted in Europe is a recent development in Iranian culture and that people’s sense of self is defined largely through conduct and situationally dependent rather than located in ‘who I am’ (2014: 297). Thus, rather than being ‘gay’ or ‘lesbian’, a young male (*amrad)* might during a particular phase of his life, have sexual relations with an older man (El-Royayheb, 2005: 42). Similarly, as Afary shows, there are accounts of ‘sisterhood’ vows between two married women that are negotiated through a ‘love broker’ and involve a period of courtship (Afary, 2009: 102). These norms changed from the nineteenth century onwards with increasing engagement with Europe and a growing heteronormative orthodoxy. As heterosexuality and gender equality came to be associated with European values, same-sex relationships between men were attributed to homosociality and the exclusion of women from public space and developed negative connotations (Korycki & Nasirzadeh, 2016: 55; Najmabadi, 2005: 3).

Corresponding to the above are differences in language and terminology. Bucar and Shirazi point out that there is no ‘exact cognate’ of homosexual in the Qu’ran and the idea that someone’s sexual desire indicates something about their identity is unfamiliar (2012: 423). Korycki and Nasirzadeh state that ‘[t]he most commonly used terms denoting homosexuals refer to sexual acts or behavioural traits. *Hamjensbaaz* is the most frequently used word, where *hamjens* means same-sex, and *baaz*, a player’ (2016:60). They describe the development of new Persian terms with positive connotations, such as *Hamjens-gara* (a person sexually oriented to the same-sex) (Korycki & Nasirzadeh, 2016: 59–60).

Moving towards the present day, the same authors describe gay identity development in Iran in terms of moments—itself indicative of the mutability of sexuality. They argue that it is only with the failed opening up of political space under the Khatami presidency ending in 2005 and the expansion of the internet as the basis for sexual relationships that the category of the gay subject—men who called themselves ‘gay’—emerged (Korycki & Nasirzadeh, 2016). This, of course, resonates with Massad’s claim that gays and lesbians in Muslim Arab society are the production of the ‘Gay International’ (2008).^5^ Mahdavi also pinpoints a shift at this time from gender fluidity to the strategic claiming of a gay identity. Prior to 2004, her interviewees rejected labels such as ‘homosexual’ and ‘heterosexual’ and any line between the two was blurred. However she attributes the change to political resistance rather than personal reconceptualisation:

Between 2005–2007, some of my interlocutors began to talk about the birth of what they called a ‘strategic gay movement’ and started discussing LGBT (Lesbian, Gay, Bisexual, Transgender) activism in Iran as a part of the sexual revolution that seeks to change discourse on sexuality as part of a push for social change in Iran. (Mahdavi, 2012: 224).

One of Mahdavi’s interviewees explains that they had ‘always enjoyed men’ but now said ‘I’m gay’, and justified this as a political act aimed at regime destabilisation (Mahdavi, 2012: 232). In this way, the fluidity of identity in Iranian history now co-exists with a rights-based discourse of LGBTQI + identity.

Iranians’ adoption of the language of LGBTQI + personhood is congruent with theories of homonationalism and homonormativity, in which the global gay rights movement is a proxy for neoliberalism (Puar, 2007; Rao, 2015). At the same time, there is an obvious convergence between Western queer theory’s now well-established deployment of performativity and Iran’s tradition of presenting sexuality as ‘what you do’ rather than ‘who you are’. However, international refugee law is not designed to capture such fluctuations and nuances in self-identification, as the next section considers.

## SOGIESC Refugee Protection

Iran is a state in which certain same-sex sexual acts are illegal and punishable by sentences ranging from imprisonment to the death penalty. The positioning of sexuality as role or activity discussed earlier persists in the existing Iranian law that penalises the ‘receiver’ more harshly than the ‘giver’ in the context of anal penetration:

The *hadd* [religiously prescribed] punishment for *livat* [male-male sex] shall be the death penalty for the insertive/active party if he has committed *livat* by using force, coercion, or in cases where he meets the conditions for *ihsan*; otherwise, he shall be sentenced to one hundred lashes. The *hadd* punishment for the receptive/passive party, in any case (whether or not he meets the conditions for *ihsan*) shall be the death penalty. (Islamic Republic of Iran, 1991: Article 234).

The Iranian state is regularly identified as one of the harshest global abusers of women and queer individuals, who are accordingly entitled to seek international protection under the Refugee Convention (Human Rights Watch, 2023; Home Office, n.d.). This article is not suggesting that queer Iranians should not be recognised as refugees. On the contrary, it is questioning whether the Refugee Convention, as interpreted, provides sufficient recognition to queer claimants.

To qualify for protection as a refugee under the Refugee Convention, an individual must show that they have a well-founded fear of persecution ‘for reasons of race, religion, nationality, membership of a particular social group (PSG) or political opinion’ (UN General Assembly, 1951). Persecution because of sex and gender is not on the list of reasons and in the immediate decades after the Convention was open for signature and ratification, there was little recognition of SGBV as the basis for asylum (Arbel et al., 2014: Introduction; Dustin, 2018: 104; Edwards, 2010: 21–22). It is only since the 1980s that the harms experienced by women, sexual and gender minorities have been recognised as the basis for international protection. A key element in bringing about this recognition was the case of *Shah and Islam* in the UK in 1999 (*Islam v. Secretary of State for the Home Department*, 1999). This appeared to make redundant debates about the desirability and viability of a new Convention ground for gender by reinterpreting one of the existing grounds. The case affirmed that women could be in need of protection by virtue of belonging to a PSG, in this case a group with the following shared characteristics: they were Pakistani women who were accused of transgressing social mores (here adultery and disobedience to their husbands), and who were unprotected by their husbands or other male relatives (*Islam v. Secretary of State for the Home Department*, 1999: 14). The case was also ground-breaking for SOGIESC-based claims in the UK in confirming that ‘homosexuals may in some countries qualify as members of a particular social group’ (*Islam v. Secretary of State for the Home Department*, 1999: 10).

In the years since, PSG has become the default ground for claims by women, sexual and gender minorities in refugee-receiving countries, with case law and international and domestic guidance steering decisionmakers on its use. The basis for qualifying for PSG membership has two elements according to UNHCR:

a particular social group is a group of persons who share a common characteristic other than their risk of being persecuted, *or* who are perceived as a group by society. The characteristic will often be one which is innate, unchangeable, or which is otherwise fundamental to identity, conscience or the exercise of one’s human rights (UNHCR, 2002: 3-4. Emphasis added).

While UNHCR provides these criteria as alternatives, EU member states and those that follow the EU’s lead, including the UK, require claimants to show both that they share a common identity and that they are perceived as a distinct group, creating a higher bar for establishing credibility than envisaged by UNHCR. While definitions of PSG have been interrogated from a number of perspectives (Marouf, 2008; Wessels, 2021, Ch 8), here I focus on the obstacles to protection created by the first criteria, the requirement of a shared common characteristic that is likely to be ‘immutable’:

a group is united by an immutable characteristic or by a characteristic that is so fundamental to human dignity that a person should not be compelled to forsake it. An immutable characteristic may be innate (such as sex or ethnicity) or unalterable for other reasons (such as the historical fact of a past association, occupation or status) (UNHCR, 2002: 2-3).

With the words ‘immutable’, ‘innate’ and unalterable’, this arm of the PSG criteria fits better with the (European/Western) focus on identity rather than the (Middle-East/Asian) behaviour-based model of SOGIESC.^6^ It is repeated or paraphrased in guidance and case law in the majority of Western and European jurisdictions, including EU Member States, the UK, the USA, Canada and Australia (Council of the EU, 2011; Home Office, 2011; US Citizenship & Immigration Services, 2021; Immigration & Refugee Board of Canada, 2017; Administrative Appeals Tribunal, Australia, n.d.).^7^ Emphasising identity as an attribute, Australian tribunal guidance states: ‘Australian Courts have emphasised that the primary focus of this Convention ground is on what a person is—a member of a particular social group—rather than what a person has done, or may do, or possesses’ (Administrative Appeals Tribunal, Australia: “Voices of queer Iranians in exile” section).

Where the ‘immutable’ nature of ‘homosexuality’ was once expected to be proven through intimate photographs and evidence of sexual relationships, now evidence of an emotional journey to the point of self-discovery on the part of the claimant is expected (Danisi et al., 2021: Ch.7; Jansen, 2019). In either case, the refugee status determination process is based on the premise that LGBTQI + individuals are a verifiably distinct kind of person and the task of the refugee decisionmaker or assessor is to undertake that verification, typically by identifying whether the individual before them is genuinely gay or a fraud.

The emphasis in decisionmaking on the claimant’s identity rather than external persecution risks is notable (Dustin & Ferreira, 2021). A 2022 survey by the European Union mapped guidance and practice in SOGIESC-based asylum, focusing on the examination of the claim. It found that:

The credibility assessment of SOGIESC claims focuses mainly on the applicant’s internal experiences. The availability of external evidence such as documentary evidence and comprehensive, detailed and reliable country of origin information is often limited (EUAA, 2022).

Despite awareness that ‘credibility assessment should not be based on Western stereotypes and assumptions on SOGIESC’, the survey suggests that elements relating to the ‘discovery of one’s own orientation/identity’ carry greater weight for decisionmakers than the ‘situation in the country of origin’ (EUAA, 2022).

The same emphasis is apparent in UNHCR guidelines which recognise that for some people sexual orientation and gender identity ‘may continue to evolve across a person's lifetime’ (UNHCR, 2012: 3). At the same time, the guidelines refer to claims from ‘members of specific sub-groups, that is, lesbian, gay, bisexual, transgender, intersex and queer individuals (usually abbreviated as “LGBT”, “LGBTI” or “LGBTIQ”)’ (UNHCR, 2012: 3). The guidelines include two pages of terminology and definitions, and while they reference ‘perception’, they focus on ‘LGBTI applicants’ before saying ‘[b]ehaviour and activities may relate to a person’s orientation or identity in complex ways. It may be expressed or revealed in many subtle or obvious ways, through appearance, speech, behaviour, dress and mannerisms; or not revealed at all in these ways’ (UNHRC, 2012: 6). This is a statement that applies to anyone, regardless of sexuality or gender identity.

The creation of such guidance is positive, particularly if drafted in consultation with people with lived experience of queer asylum and their advocates. All the guidance read for this article stresses the need for sensitivity and the avoidance of stereotyping and assumptions in interviewing and decisionmaking. However, despite the recognition that persecution is based on *perceptions* about the individual (eg Immigration & Refugee Board of Canada, 2017: para 8.3.1), the focus is almost exclusively on the fairest way of establishing whether someone definitively *is* or *is not* queer. Canada’s guidance refers to the ‘challenges faced by SOGIESC individuals in establishing their SOGIESC’ (2017: para 3), stating that ‘[t]estimony about same-sex relationships that is vague and lacking in detail may support a negative credibility inference’ (2017: para 7.6.1). The assumption here is that SOGIESC asylum claimants are SOGIESC minority individuals (rather than imputed to be so) and that engaging in same sex relationships—and being able to offer detailed and rich accounts of those relationships—is one of the best proofs of that fact.

In this section, I have highlighted the homogenising assumptions about sexuality, gender and gender identity that underpin refugee law and guidance on decisionmaking in a number of jurisdictions.^8^ Despite making important recommendations, for example relating to the need for sensitivity and the difficulties claimants may face in speaking openly, the overwhelming focus of guidance is on supporting decisionmakers, who are more likely to have a background in law than in sexual or behavioural psychology, in categorising the claimant on the basis of SOGIESC and without recognising the cultural-specificity of such a determination process. In the section that follows, I share the voices of some participants in the NQIfFM project to consider the extent to which refugee assessment criteria corresponds to lived experiences to indicate the extent to which SOGIESC asylum claims are assessed on a fair basis.

## Voices of Queer Iranians in Exile

In this section, I draw on the contributions of our project participants to evaluate the consistency between how a group of exiled queer Iranians talk about their sexuality and gender identity and how they—and other asylum claimants—are expected to do so in refugee status determination.

### Language and Terminology

A first consideration relates to simple language distinctions and more subtle cross-cultural differences in terminology and conceptualisation. Asylum claimants are required to present their case in ways that are intelligible to Western decisionmakers who may or may not have a legal training but are unlikely to have studied sexuality and gender. Iranian claimants in our research explained that they often found it confusing to have to define themselves using terms that did not exist in Farsi. Alireza needed to search on the internet in English, not Farsi, when they were looking for articles about being queer. They only engaged with the language of identity on leaving Iran and here appear to view it as a foreign language to be learned:

Well, when I came here [to the UK], I identified as gay. There was not much information available in Iran, it was a restricted space. Although I read a lot, I knew maybe about three or four identities. It was when I moved here that I realised how huge, extensive and multiple it can be.

They first encountered the term ‘non-binary’ through the English TV series ‘Shameless’, which had a non-binary character: ‘it immediately clicked: “Oh! They look so much like me!”’.

Others talked about the encounter with unfamiliar descriptors such as LGBT. Karen explained how ‘via the internet, [he] became acquainted with terms such as gay, lesbian, homosexual, LGBT, although back then LGBT was not so much in use (…)’. Amir also relied on the internet: ‘Because at that time, I couldn’t pose these kinds of questions with my sexual partners and ask: “What is our relationship called?” or “What is your identity and my identity?”’.

Amira found that ‘language matters a lot’ in relation to gender when they were asked about their gender identity in a job interview:

And then I felt, for the sake of the English language, I have to accept something that hasn’t been my problem so far. They [the interviewers] got confused as well. So, I explained to them that in Farsi we do not have ‘she’ or ‘he’, or even ‘they’. We use a single *ou* for everyone. And I have always been the *ou*. Now I have to choose between ‘she’, ‘he’ and ‘they’.

In a similar way, Zeynab describes being asked what their pronoun was by members of a Swedish collective they were a part of, and their response was: ‘Why on earth people should have gender pronouns? I couldn’t understand it. I was saying, “call me ‘they’ because we don’t have gender pronouns in Persian”’. Zeynab went on to describe sex and gender in terms of an ‘indefinite/unlimited spectrum’ that cannot be explained. They asked themselves ‘why on earth am I using so many labels. Maybe queerness expresses them all’, and ultimately settled for ‘queer’ as a descriptor, albeit an unsatisfactory one. Although they had used different labels throughout their life—including ‘gender fluid’, ‘non-binary female’ and ‘multi-gender’, they felt that describing themselves using one term inevitably meant losing another aspect of their identity:

Even nonbinary is not enough for me. When introducing myself as non-binary, I feel I ignore another part of my identity to be fitted into that framework, in order to be enough nonbinary and enough queer. It is like introducing myself as a homosexual. In that case, I ignore my fluidity by pointing out one part of it. Nonbinary is the same.

Many participants disliked the expectation that they should fit into categories or, alternatively, found the categories available inadequate. In this respect, Hope said: ‘I don’t really give a shit if they call me “they” or “she” … and often I don’t even wish to define a gender for myself.’ Jebo was made uncomfortable by the expectation that they categorise themselves: ‘I am like this, and why should there be a name and category for it’. Raha also said ‘I changed my gender from female to male when I was 18. However, I am not concerned with whether I am a woman or a man, and it is not important to me, it’s not a big deal.’

There is a congruence between the rejection of labels by some of our participants and the development of a global discourse embracing ‘queer’ as an inclusive but at the same time unstable identifier. Farhan told us: ‘Many years ago, I had read about generation X and non-binary and already then I realised that I belong to that category in that I do not belong to any of the two [male or female] and that I wished to be myself beyond categories.’ Alireza did not agree with this kind of labelling either, but at the same time said that ‘I would like to identify myself. I mean, I would like to know which group I belong to.’ In language recognisable across queer discourse, Amira described themselves as ‘a transgender woman, somewhere between a transwoman and a nonbinary or genderless.’ Likewise, Darya described themself as ‘bisexual and pansexual’ but also told us ‘I am gender fluid, but lean towards “she”’.

It may be the case that the historical rejection of fixed sexual and gender identities in Iran is now reflected in broader trends in queer politics, literature and discourse that reject stable identities (Ahmed, 2016; Barker & Iantaffi, 2019; Hines, 2020). If so, alongside this phenomenon, movements through space and over time are also a factor informing how people identify, as the next section discusses.

### Time and Place

The categories within the LGBTQI + acronym and its variations were misaligned with our participants’ identities in ways beyond those explored above: for many of them, there was a change in self-awareness over time and as they moved from one country to another, from Iran to Turkey, and then to either the UK or Canada. For a number of people, the need to think about and articulate a sexual or gender identity only happened after leaving Iran. Sobhan, for example, explained that it was only in the previous two years that they had come to identify as non-binary through research, self-exploration and counselling: ‘The journey of self-discovery is a transformative process, and I underwent changes in time, place, and circumstances while transitioning between these three countries [Iran, Turkey and Canada]’.

Pedran did not explore their identity, even in Turkey:

The topic of gender identity did not get tangible for me until I arrived in Canada. When I was in Turkey (…) I had so much tension, crisis and emergencies that I did not have the space or the capacity to reflect or get deeper into such topics (…). I could open that door and say ‘Alright, now I can have a second look at this topic’ only when I arrived in Canada and could settle more and reach more peace.

Clover, on the other hand, did experience a change in self-perception in Turkey, ‘when I realized that I did not want to identify as gay anymore but as a “gender queer” person with queer sexual orientation.’

People’s identities changed not only as they relocated, but over time and depending on their age and generation. On this point, Alireza said:

In the beginning, I thought I was a transgender, but later I realized it did not match me much. So, I mostly think I have a non-binary or a gender that cannot be reduced to one of the binary ones that exist. I do not have any tendency to want to identify myself as a man or a woman.

In Darya’s twenties, gender had been important to their identity but that was no longer the case: ‘Agender or maybe, better, gender fluid is what I see myself as’, but they also said precise gender labels were not important to them.

Those who had left Iran some years ago rather than more recently observed a generational difference for young queer people who had access to the internet and social media in a way that they had not. Zeynab, for example, said:

I am always astonished to see the queer attitudes among Iranian youth in social media and their complicated understanding about their sexual orientation and gender identity. Sometimes, I feel I cannot understand what they are saying, and I need to search more. To educate myself to catch up with them. They look very similar to teenagers living here [in the UK].

Likewise, Homa said that ‘[t]he younger generations here [in Canada] are very open and comfy, whether with being gay themselves or the queerness of others.’ Shaya captured this shift in self-identification, including the relevance of social media developments in stating:

I think that, for my generation [Shaya was 37 at the time of interview], being confronted with gender or sexual identity and/or orientation has not been an easy task. Social media did not exist then. There were no smartphones. The generations now are more aware. Kids who are queer and live in Iran know much more, many more concepts and distinctions compared to us who live here. They know for instance that they are ‘gender fluid’, or a ‘non-binary trans’, ‘homoflexible’, ‘heteroflexible’, and this is really good. It is great that these very [queer] kids/guys have learned these subtle distinctions, because I am sure no medics in Iran could tell these to them.

This last point, the conscious rejection of fixed binaries, was a recurring one, as the next section explores.

### Fluidity

Our participants had very many different perspectives about their own sexuality and gender identity, and about the sexuality and gender discourses in the societies they had left and joined. A common thread to these perspectives was the belief that sexuality and gender are not fixed. Since the age of 18, Zeynab—mentioned earlier—has identified themself in different ways at different times:

Gradually, I identified myself as a pansexual as well as queer. It has also been a couple of years that I identify myself as one who is gender fluid, as one who is sometimes non-binary and other times a woman. I have always this feeling that I am not enough gay, enough gender queer, enough gender fluid. And I’m always out of the gender boxes when it comes to binaries.

Zeynab was not alone in their rejection of essentialism. Sima also said: ‘I don’t deem identity as an essential and unchanging thing. Our bodies and minds are changing continuously. I see these very fluidly. I am really more feminine some days as opposed to others.’ Meanwhile, Sobhan told us that ‘I’m gay. I’ve always been that way. But I believe that everything in this world is subject to change, and sexual orientation is not something fixed.’ Artin elaborated on this theme by explaining that ‘the “I” ten years ago is really different to the “I” today. (…) So, in ten years from now that person can change again.’ Finding a metaphor to express this, Pedram said: ‘I think the main shift for me was that I saw the wave-like nature of it [sexual orientation], that it was not as constant as I used to think it was, that it could move, open or close’.

This belief in fluidity, however, was not universal. Arshia said ‘I describe myself as gay. My gender identity is male, and my sexual orientation is gay. I figured it out when I was like 15 or 16’ when he began using Yahoo Messenger to connect with the gay community. For Arshia, embracing a stable gay identity was positive: ‘When I researched and realized that I wasn’t the only gay person and that there are others who are LGBTQ, I became much more comfortable accepting my identity.’ Arshia’s choice may be for strategic reasons, to engage with a like-minded community or as a political statement, but it is also simply a reflection of differences in human self-conception and related language choices.

In highlighting some of the diverse and sophisticated ways in which queer Iranians talked about their own identities and interrogated sexual and gender identity categories, these accounts highlight the dissonance between the experiences of queer exiled Iranians and the constructions of sexuality and gender identity for asylum purposes. The next section summarises this misalignment and its implications.

## Asylum Determination and Lived Experience

As shown in the section on SOGIESC Refugee Protection, asylum law, and the policy and practice that stems from it, relies on putting people into ‘boxes’. As Zeynab says:

[T]he whole idea of the asylum system is about gate keeping. It is about identifying who is telling the truth, and it is about saving a minority group of people who are in danger and deserve to be saved. So, the asylum system essentially needs clear cut definitions, essentially needs to put people into boxes.

This requirement to fit into the LGBTQI + box is incompatible with the narratives of many of our participants who describe their sexuality and gender identity in ways that are elusive and deliberately avoid easy categorisation. This does not mean they are not at risk of persecution on the basis of sexual orientation and gender identity, rather that it is inappropriate for potentially life-or-death decisions about protection to be based on a foreign legal interlocutor’s ability to establish the ‘truth’ about a claimant’s SOGIESC (Ferreira, 2022).

While the requirement for decisionmakers to establish facts as the basis for granting or refusing protection is necessarily in tension with conceptions of identity and SOGIESC as porous, the particular way that SOGIESC asylum had evolved through the application of the PSG is a further and specific problem. Often, the claimant must convince the decisionmaker of their true sexual identity as the basis for establishing membership of a social group composed of others who share that identity. While, and rightly, this is no longer done in some jurisdictions by intrusive questioning about sexual partners, an alternative approach has emerged: the tendency now is to require an account of an emotional journey to self-discovery, where an individual who has always felt ‘different’ realises that fact and explains so to the decisionmaker (Dustin and Ferreira, 2021; Jansen, 2019). This may be a more sensitive approach but it fails to reflect lived realities—both those of our participants and trends towards fluidity in queer activism and discourse globally.

The outcome is SOGIESC Refugee Status Determination (RSD) that relies on narrow and confining categories, while identity is—and is increasingly recognised as—expansive, intersectional and mutable. There is a striking incongruence between the clinical pigeonholing of sexual and gender identities that characterises asylum, and the poetic language of Iranians who participated in our research, such as Amira, who describes identity using a metaphor for cyclical reincarnation: ‘I see myself as Attar’s [Attar of Nishapur] Phoenix (…) Because that one rose from its own ashes and it’s really tough to rise from your ashes every time’.

The contradiction between the categorical nature of asylum law and the increasingly fluid nature of individual sexuality and gender identity may not be fully reconcilable and certainly merits further research. One solution may be for decisionmakers to focus less on ‘who you are’ and more on ‘is your “professed self” likely to result in persecution?’. As I have argued elsewhere (Dustin and Ferreira, 2021), decisionmaking that prioritises country of origin information is less likely to default to fruitless attempts to determine a claimant’s ‘true’ identity.

## Conclusion

This article builds on previous work that confronts the absurdity of basing refugee protection on establishing fixed ‘truths’ about sexuality and/or gender identity. Contrasting the lived experiences of a diverse group of queer exiled Iranians with the way that sexuality and queerness has come to be conceptualised within refugee status determination suggests that asylum adjudication is at odds with many people’s lived experiences. As a caveat, not all these individuals we interviewed were or are seeking asylum, and Iranian sexuality and gender have developed in a very particular socio-political and cultural context. However, the disparity between the two paradigms explored here is striking. While decisionmakers often expect claimants to have come to a realisation of their sexuality as an innate attribute, recent interviews with exiled queer Iranians suggest that many individuals experience and express their sexuality and gender very differently, as fluctuating over time and in different situations. Najmabadi convincingly demonstrates that in Iran, sexual and gender self-cognition takes place less through affirmations of identity than through notions of conduct that are context-specific, highlighting the performative and flexible nature of identity. Similarly, for the people we spoke with, SOGIESC was an important part of their identities and lives, and for many, the reason or part of the reason for leaving Iran. However, it was not the stable identifier of much Western discourse.

In light of this dissonance, the fairness and validity of approaches to RSD for Iranian queer claimants needs questioning, recognising that ‘advocacy for Iranians who do not fit a heteronormative model may not be as simple as asserting a universal gay or lesbian subject’ (Bucar & Shirazi, 2012: 417). Here, the need to define and categorise individuals that is inherent in legal decisionmaking hampers the efficacy of international protection for queer claimants because it means decisionmakers are looking for a particular narrative and a truth which may not exist regardless of whether the claimant is at risk of persecution or not. As argued here, part of the problem is the default application of the PSG Convention ground in SOGIESC asylum cases, which requires the persecuted individual to show they have a specific identity as a member of a group based on that shared identity. This expectation is increasingly at odds with the way that many individuals—asylum claimants or not—experience and articulate their sexuality and gender. While there is now some recognition in academic and public discourse of gender and sexual fluidity, this is inadequately reflected in refugee law and practice, with troubling implications for Iranian and other queer asylum claimants. By mapping the experiences of some queer Iranians in exile, this article provides further evidence of the need to rethink the basis for refugee protection from homophobia and transphobia. Beyond the field of refugee law, it contributes to the ongoing project of queer theory in disrupting homogenising discourses of identity that fail to correspond to many people’s lived experiences.

## Data Availability

There are no data associated with this article.

## Footnotes

1 Ethics approval code: ER/NF213/3.

2 Full details of the project methodology and all project outputs are at https://iranqueerefugee.net/

3 Variations of lesbian, gay, bisexual, trans, queer, intersex. See for example https://www.rainbowmigration.org.uk/what-we-do/ and https://ilga.org/about-us/

4 Sexual orientation and gender identity, or sexual orientation, gender identity and expression and sex characteristics eg https://www.sogica.org/en/; EUAA, 2023; Acronyms and abbreviations, UNHCR, n.d.

5 Massad’s argument has been found problematic by scholars including Rao and Kapur (Rao, 2015: 42; Kapur, 2018: 67–68).

6 I recognise that here I am, for the purposes of argument and length, deploying the kind of simplistic East–West binary I criticise elsewhere.

7 The difficulty that judges face in allocating a PSG as the basis for recognising queer refugees can be seen in the positive appeal decision of Mx where a Salvadorean individual’s social group changed from ‘homosexual men’ to ‘transgender women’ (*Mx M (gender identity – HJ (Iran) – terminology)* [2020] UKUT 313 (IAC) para 39).

8 See also: Bennett and Thomas 2013; Berg and Millbank 2009; Berlit et al., 2015; Fernandez 2017; Jansen 2019; LaViolette 2015.
